# Gastrointestinal Digestion Enhances the *In vitro* Antitumor Activity of Red Grape Anthocyanins Against Colorectal Cancer Cells

**DOI:** 10.1007/s11130-026-01498-w

**Published:** 2026-04-14

**Authors:** Daniel Cruceriu, Denisa Dobos, Stefan Miron, Oana Baldasici, Loredana Balacescu, Ovidiu Balacescu, Zorita Diaconeasa

**Affiliations:** 1 Department of Genetics, Genomics and Experimental Pathology, The Oncology Institute “Prof. Dr. Ion Chiricuta”, 34-36 Republicii Street, Cluj-Napoca, 400015 Romania; 2 Department of Molecular Biology and Biotechnology, “Babes-Bolyai” University, 5-7 Clinicilor Street, Cluj-Napoca, 400006 Romania; 3 Faculty of Food Science and Technology, University of Agricultural Science and Veterinary Medicine, 3-5 Calea Manastur Street, Cluj- Napoca, 400372 Romania

**Keywords:** Red grape anthocyanins, INFOGEST digestion, Colorectal cancer, Apoptosis, Cell cycle arrest, Cell migration, Transcriptomics

## Abstract

Anthocyanins from red grapes possess notable anticancer properties, yet their biological activity after gastrointestinal digestion remains poorly characterized. In this study, we investigated the in vitro antitumor effects of a red grape anthocyanin extract digested using the INFOGEST protocol on colorectal cancer (CRC) cell lines. The study assessed cell viability, apoptosis induction, cell cycle progression, and confined 3D migration, and integrated whole-transcriptome microarray profiling to elucidate the underlying molecular mechanisms.Cell viability assays showed that simulated digestion markedly increased the extract’s potency, reducing the IC_50_ values by 2.25-fold in DLD1 cells and 1.59-fold in Caco2 cells. Transcriptomic profiling by microarray analysis demonstrated substantial gene expression changes upon treatment in DLD1 cells, with 873 genes showing a fold regulation greater than |±1.5|. Gene Set Enrichment Analysis (GSEA) identified enrichment of pro-apoptotic gene sets in treated cells, including p53 signaling, prolonged unfolded protein response activation and suppression of RNA metabolism. These findings were supported by the Annexin V/PI assay demonstrating apoptosis induction, and RT-qPCR validation of key apoptosis-regulating genes. The extract also displayed significant antiproliferative activity with cells arrested in the S phase, as evidenced by AlamarBlue and cell cycle analyses. Downregulation of key cell cycle promoters and upregulation of proliferation-inhibitory genes were also observed. Furthermore, the extract significantly impaired 3D migration in a microfluidic model, reducing migration speed, directionality, and number of migratory cells. GSEA revealed suppression of RHO GTPases and MIRO-mediated signaling, suggesting disruption of cytoskeletal and mitochondrial dynamics, strongly impacting cell migration. Together, these findings indicate that digested red grape anthocyanins exert a multifaceted anticancer effect in vitro by promoting apoptosis, inhibiting proliferation, and impairing cell migration, highlighting their potential as functional bioactive compounds for CRC management.

**Supplementary Information:**

The online version contains supplementary material available at 10.1007/s11130-026-01498-w.

## Introduction

Anthocyanins are water-soluble flavonoid pigments widely distributed in plant tissues [1], recognized for their diverse biological activities, including antioxidant, anti-inflammatory, antimicrobial, and cardioprotective properties [2]. Among anthocyanin-rich sources, red grapes stand out for their high anthocyanin content, predominantly in monoglucoside form. Malvidin-3-glucoside is typically the most abundant compound, and this specific glycosylation pattern significantly influences stability, absorption, and biological activity [3], positioning red grape anthocyanins as promising candidates for functional foods and nutraceuticals aimed at disease prevention.Anthocyanins have also shown considerable promise in both cancer prevention and therapy. In vitro and in vivo studies have demonstrated that anthocyanins can modulate key pathways involved in tumor development [4]. In colorectal cancer (CRC), the third most commonly diagnosed cancer worldwide [5], dietary factors are known to significantly influence disease onset and progression [6]. Within this context, anthocyanins have emerged as chemopreventive and therapeutic agents with great potential in CRC management. They have been reported to regulate oxidative stress and inflammation, induce apoptosis, arrest cell cycle progression, and inhibit invasion and angiogenesis in CRC models [6]. Red grape anthocyanins, in particular, have demonstrated selective cytotoxicity toward colon cancer cells while sparing normal colon epithelial cells [7], supporting their potential use in targeted prevention or treatment strategies.However, a major challenge in translating these findings to clinical relevance lies in anthocyanin bioavailability. Anthocyanins are relatively unstable in the gastrointestinal tract and undergo extensive transformation during digestion, reaching the colon primarily as diverse metabolites rather than parent compounds [8]. Simulated gastrointestinal digestion models have therefore become indispensable for evaluating anthocyanin bioactivity under physiologically relevant conditions [9], particularly for CRC, where the colon itself is a direct site of action.

In this study, we aimed to evaluate the in vitro antitumor activity of a red grape anthocyanin extract subjected to simulated gastrointestinal digestion using the INFOGEST protocol. Using human colorectal cancer cell lines, with a focus on DLD1 cells, we examined the extract’s effects on cell viability, apoptosis, cell cycle progression, and confined 3D migration. Furthermore, we integrated transcriptomic profiling to uncover the molecular mechanisms underlying the observed cellular effects. By simulating digestion, this work provides a insights into the functional relevance of anthocyanins in a more physiologically relevant form, contributing to the ongoing efforts to validate dietary compounds for colorectal cancer chemoprevention and treatment.

## Materials and Methods

### Anthocyanin Extract Preparation

Anthocyanins were extracted from red grape skins (*Vitis vinifera*) using acidified methanol and purified by solid-phase extraction (SPE) on C18 Sep-Pak cartridges, as previously described [4].

### Static *In vitro* Digestion and Purification of the Anthocyanin Extract

The purified extract was subjected to simulated gastrointestinal digestion following the INFOGEST 2.0 static protocol [9]. Description of the protocol can be found in SM.

### Phytochemical Characterization of Anthocyanin Extracts Using LC-DAD/ESI^+^-MS

Phenolic compounds were analyzed using an Agilent HP-1200 HPLC system (Agilent Technologies, CA, USA). The LC-DAD-ESI⁺/MS method is described in SM.

### Cell lines and Culture Conditions

Two human colorectal carcinoma cell lines, Caco2 (ATCC, USA) and DLD1 (ECACC), were cultured under standard conditions as detailed in SM.

### Evaluation of the *In vitro* Antitumor Activity by the MTT Assay

Cell viability was assessed by MTT assay (#M6494, Invitrogen) on Caco2 and DLD1 cells treated for 24 h with serial dilutions of undigested (5–40 µg/mL) and digested (1–25 µg/mL) extracts. IC₅₀ values were determined by nonlinear regression (GraphPad Prism 8). Detailed procedures are described in SM.

### Functional Validation Assays

The antitumor activity of the digested extract was further characterized in DLD1 cells at IC₅₀ and 5 µg/mL concentrations using Trypan Blue exclusion, Annexin V/PI flow cytometry, AlamarBlue proliferation kinetics, and cell cycle analysis by propidium iodide staining. Detailed procedures for all assays are described in SM.

### Evaluation of Cell Migration in 3D Microfluidic Devices

Confined 3D migration was assessed using microfluidic devices containing 10 μm-wide.

channels [10]. DLD1 cells treated with IC₅₀ or 5 µg/mL were monitored by time-lapse microscopy, and migration parameters were quantified using ImageJ. Detailed procedures are described in SM.

### Transcriptome Analysis by Microarray Expression Profiling

Whole transcriptome profiling was performed using Agilent 4 × 44k microarrays. Differential expression was analyzed with limma [11], (|FR| ≥ 1.5, adj. *p* < 0.05) and GSEA [12] (FDR < 25%, nominal *p* < 0.05). Data are available in NCBI GEO (GSE307005). Detailed procedures are described in SM.

## Gene Expression Analysis by RT-qPCR

Microarray results were validated by RT-qPCR using the ΔΔCt method [13] with RN18S1 as reference gene. Primer sequences are provided in SM Table 1.

## Results and Discussion

### Phytochemical Characterization of Undigested and Digested Red Grape Anthocyanin Extracts

HPLC analysis of anthocyanins extracted from red grapes revealed nine major compounds, derivatives of delphinidin, cyanidin, malvidin, peonidin and petunidin, with a total concentration of 1016.4 µg/mL (Table 1). After in vitro gastrointestinal digestion, the total anthocyanin content decreased sharply to 322.8 µg/mL, representing a 68% reduction, with delphinidin-3-O-glucoside no longer detectable (Table 1, SM). In parallel, eight phenolic acid metabolites were identified exclusively in the digested extract, accounting for a combined concentration of 607.6 µg/mL. The most abundant metabolites were protocatechuic acid (110.4 µg/mL), gentisic acid (101.4 µg/mL), and gallic acid (92.1 µg/mL), characteristic degradation products of anthocyanin catabolism.

### *In vitro* Antitumor Activity of Undigested and Digested Red Grape Anthocyanins

**Fig. 1:**
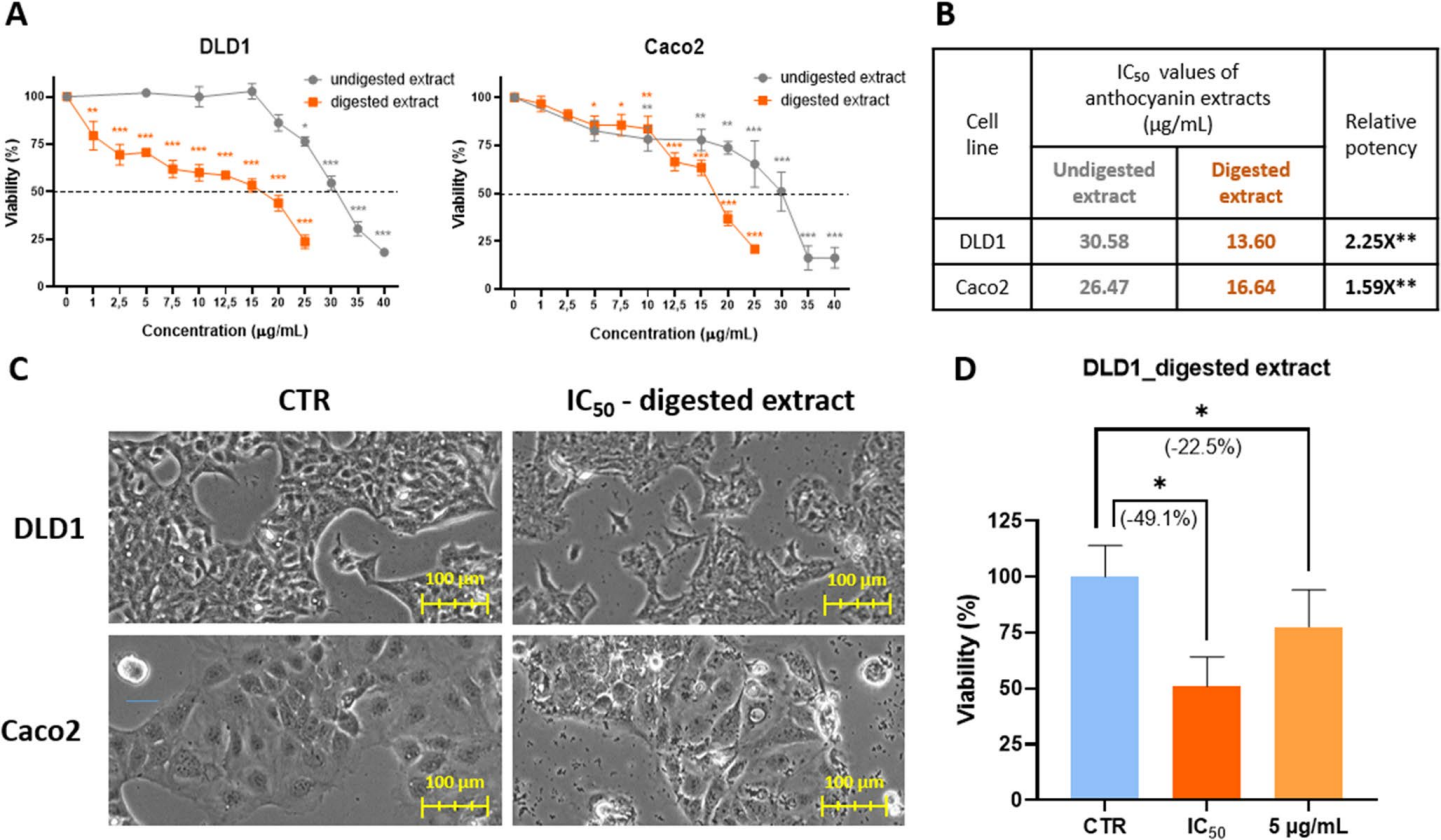
In vitro antitumor activity of undigested and digested red grape anthocyanin extracts in DLD1 and Caco2 cells. (**A**) MTT cell viability curves; (**B**) IC₅₀ values and relative potency; (**C**) Representative cell morphology of untreated (CTR) and IC₅₀-treated cells; (**D**) Trypan Blue viability validation in DLD1 cells. Statistical significance was assessed by t-test and Extra Sum-of-Squares F Test

The antitumor potential of both undigested and digested anthocyanin extracts from red grapes was evaluated in vitro using DLD1 and Caco2 colorectal cancer cell lines by the MTT assay (Fig. 1A). The undigested extract exhibited antitumor effects in both cell lines, with IC_50_ values of 30.58 µg/mL for DLD1 cells and 26.47 µg/mL for Caco2 cells. Following simulated gastrointestinal digestion, the antitumor activity of the extract was markedly enhanced, resulting in a 2.25-fold increase in potency in DLD1 cells and a 1.59-fold increase in Caco2 cells (Fig. 1B). This enhancement was statistically significant, as determined by the Extra Sum-of-Squares F Test comparing the dose–response curves and IC_50_ values of the undigested and digested extracts. In addition to reducing cell viability, treatment with the digested extract at the IC_50_ concentration also induced notable morphological alterations in both cell lines (Fig. 1C). Based on the greater relative potency observed in DLD1 cells, this cell line was selected for subsequent experiments.

To confirm the in vitro antitumor activity of the digested extract, a Trypan Blue exclusion assay was performed as an independent, complementary viability assessment. Treatment with the IC_50_ concentration, as determined from the MTT assay, led to a 49.1% reduction in viable DLD1 cells in the Trypan Blue assay, yielding results that were highly consistent and superimposable between the methods (Fig. 1D). Additionally, a lower concentration (5 µg/mL) was included to assess dose-dependent effects, as it corresponded to approximately 75% viability in the MTT assay. Consistently, treatment with this concentration resulted in a 22.5% decrease in viability in the Trypan Blue assay. Based on these findings, the IC_50_ (13.6 µg/mL) and 5 µg/mL concentrations of the digested extract were selected for further functional analyses.

Red grapes are a rich source of polyphenolic compounds that have garnered significant attention for their antitumor potential, particularly in the context of colorectal cancer (CRC), where dietary factors are known to substantially influence disease onset and progression [6]. Among these polyphenols, red grape anthocyanins have emerged as the most potent antitumor agents, exhibiting stronger bioactivity than other classes such as phenolic acids, flavonols, and other flavonoids.

The anthocyanin profile of the undigested extract is consistent with previous reports on *Vitis vinifera* varieties, though some structural variations may depend on cultivar type and viticultural conditions. Importantly, anthocyanin-rich extracts with similar compositions derived from various red grape sources have demonstrated chemopreventive efficacy in multiple in vivo CRC models and strong in vitro antitumor activity across several CRC cell lines [14, 15], underscoring the therapeutic potential of red grape anthocyanins in colorectal cancer. Nevertheless, a key limitation of most studies is that they assess the effects of native anthocyanins, which have low bioavailability and undergo extensive degradation during gastrointestinal transit [16]. In agreement with this, our simulated digestion model revealed a marked decline in anthocyanin concentration, including the complete loss of delphinidin-3-*O*-glucoside, consistent with similar degradation patterns reported for grape juice and wine [14, 17]. High levels of protocatechuic acid and *p*-hydroxybenzoil derivatives were detected after digestion, consistent with previous reports identifying them as common downstream products of anthocyanin catabolism. Since local antitumor effects within the colon are shaped by these metabolites rather than by native anthocyanins [8], their biological activity is of particular relevance. Indeed, metabolites generated through colonic microbiota metabolism [18, 19] and gastrointestinal digestion [18, 20] of red grape polyphenols have demonstrated genoprotective and antitumor effects in CRC cell lines, with extract potency increasing by approximately 25% after digestion [18]. Our findings complement these data, providing the first evidence of the in vitro antitumor activity of red grape anthocyanin metabolites generated through gastrointestinal digestion in colorectal cancer cells, with digestion enhancing anticancer efficacy to an even greater extent than previously reported for whole polyphenolic fractions [18].

### Mechanistic Insights into the Antitumor Effects of Digested Red Grape Anthocyanins

**Fig. 2:**
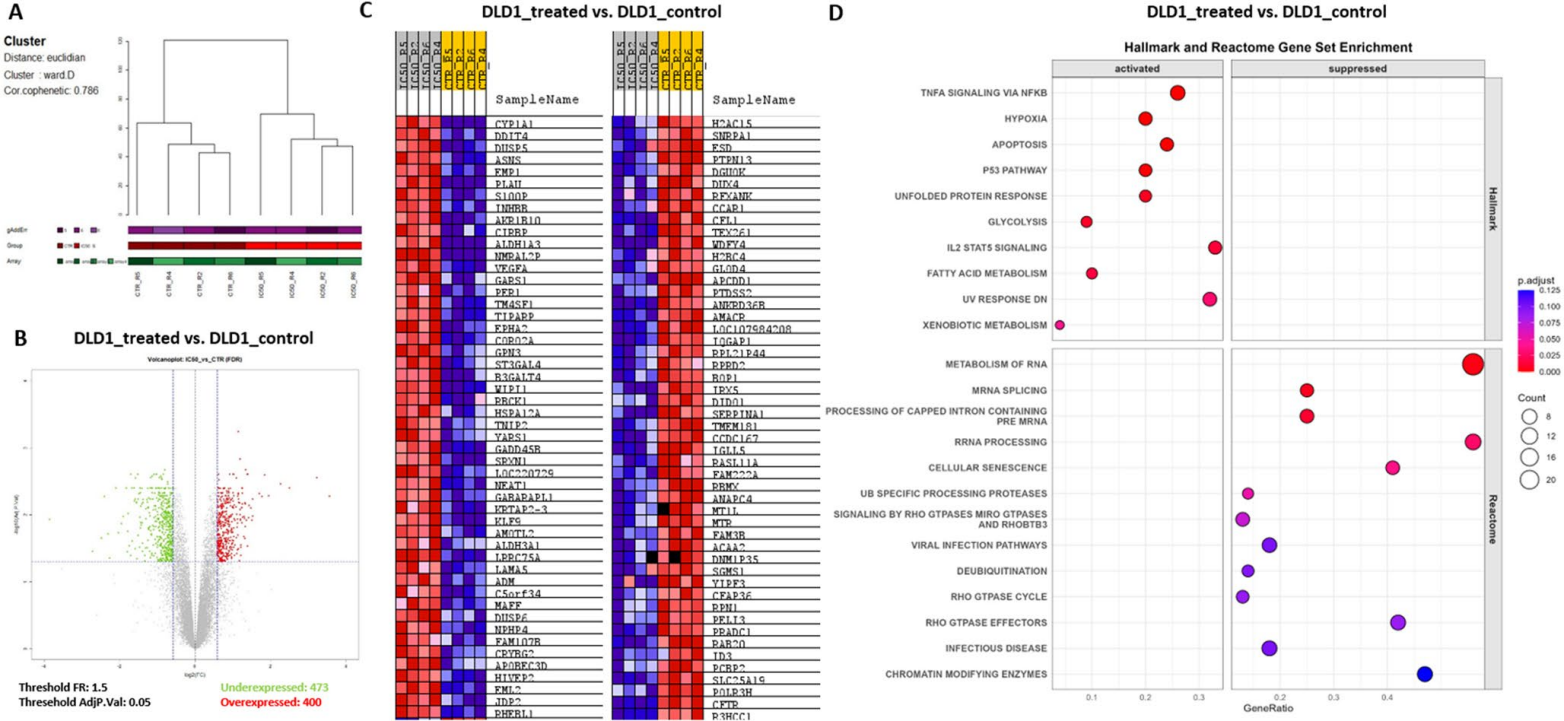
Transcriptomic alterations in DLD1 cells treated with IC₅₀ of digested red grape anthocyanins. (**A**) Hierarchical clustering of microarray samples; (**B**) Volcano plot of differentially expressed genes (|FR| ≥ 1.5; adj. *p* < 0.05); (**C**) Heatmap of top 50 up- and downregulated genes; (**D**) Enriched Hallmark and Reactome gene sets identified by GSEA (FDR < 25%, nominal *p* < 0.05)

To investigate the molecular mechanisms of action of the digested red grape anthocyanin extract, a microarray-based whole transcriptome analysis was performed on DLD1 cells treated with the IC_50_ concentration vs. untreated controls, using four biological replicates. Hierarchical clustering showed distinct separation between groups (Fig. 2A), with 873 out of 1,917 significantly differentially expressed genes (adj. *p* < 0.05) showing |FR| > 1.5 (Fig. 2B and C). GSEA revealed significant enrichment of 10 Hallmark gene sets in treated cells and 13 Reactome gene sets in controls (Fig. 2D), primarily associated with apoptosis, cell migration, and cellular stress responses, as detailed below.

### Digested Red Grape Anthocyanins Induce Apoptosis in DLD1 Cells

**Fig. 3:**
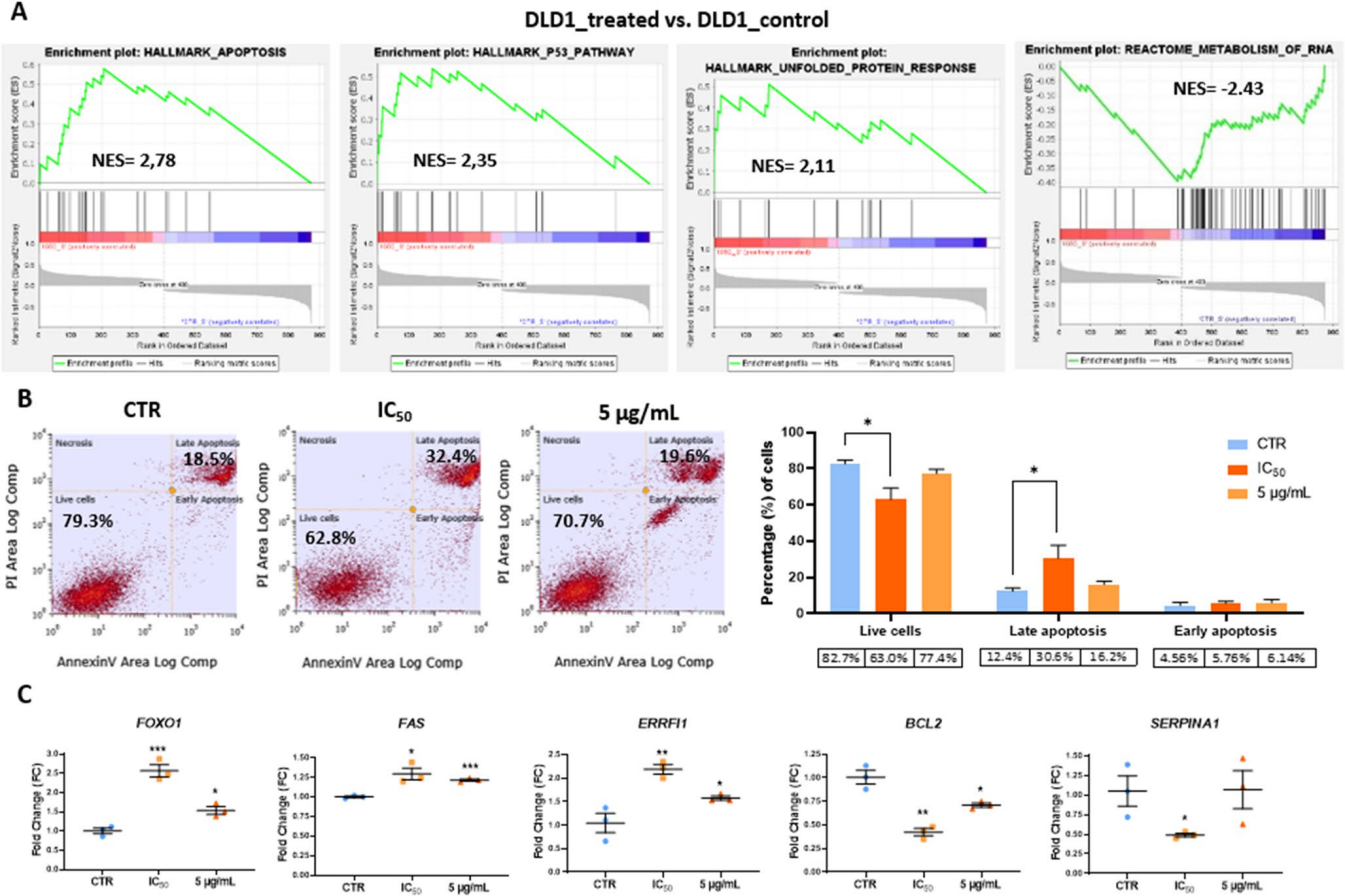
Apoptosis-related effects of digested red grape anthocyanins in DLD1 cells. (**A**) Enriched apoptosis-related gene sets (GSEA); (**B**) Annexin V/PI apoptosis quantification at IC₅₀ and 5 µg/mL; (C) RT-qPCR validation of selected apoptosis-associated genes. *p < 0.05, **p < 0.01, ***p < 0.001 (t-test)

GSEA analysis based on microarray data revealed significant enrichment of the apoptosis gene set in DLD1 cells treated with the IC_50_ concentration of the digested red grape anthocyanin extract, with a Normalized Enrichment Score (NES) of 2.78 compared to untreated controls (Fig. 3A). In addition, the pro-apoptotic p53 signaling pathway was also found to be activated following treatment (Fig. 3A). Consistent with the activation of apoptotic mechanisms, downstream cellular responses, including sustained activation of the unfolded protein response and suppression of RNA metabolism and processing, were also enriched in treated cells (Fig. 3A).

Anthocyanins are known to induce apoptosis engaging both intrinsic and extrinsic pathways, and red grape-derived anthocyanins have been shown to promote apoptotic cell death in CRC models, although the underlying mechanisms remain poorly defined. Undigested red grape polyphenolic extracts and digested grape pomace have previously demonstrated pro-apoptotic effects in CRC models, primarily through modulation of the BAX/BCL2 ratio, IAP downregulation, p53 activation, and caspase-3 upregulation [15, 20–23]. In this study, we show that the pro-apoptotic activity of red grape anthocyanins is preserved after gastrointestinal digestion, with key mechanisms mirroring those observed for whole polyphenolic extracts. Transcriptomic analysis revealed activation of the p53 signaling pathway and modulation of genes involved in both intrinsic and extrinsic apoptotic cascades. Notably, the FAS death receptor and its transcriptional regulator FOXO1 [24] were upregulated, while BCL2 [25] and SERPINA1, which counteracts apoptosis via the PI3K/AKT pathway [26], were downregulated. These molecular changes were accompanied by sustained activation of the unfolded protein response and repression of RNA metabolism, further supporting apoptosis induction by the digested extract. Notably, other studies have shown that red grape polyphenolic extracts can also activate caspase-independent apoptotic mechanisms [23], expanding the potential modes of action that may be preserved after digestion.

### Digested Red Grape Anthocyanins Inhibit Proliferation in DLD1 Cells

The proapoptotic effects of the digested anthocyanins from red grapes were confirmed by the Annexin V/PI flow cytometry assay, which demonstrated a significant induction of cell death in DLD1 cells, primarily through apoptosis. Following treatment with the IC_50_ concentration, approximately 36% of cells were in early or late apoptotic stages, compared to 17% in untreated controls (Fig. 3B). Even at a lower concentration (5 µg/mL), the extract induced apoptosis in 22.3% of cells. However, this increase did not reach statistical significance (Fig. 3B).

RT-qPCR validation confirmed the upregulation of pro-apoptotic genes (FOXO1, FAS, ERRFI1) and downregulation of anti-apoptotic genes (BCL2, SERPINA1) at the IC_50_ concentration, with dose-dependent effects persisting at 5 µg/mL for all genes except SERPINA1 (Fig. 3C).

**Fig. 4:**
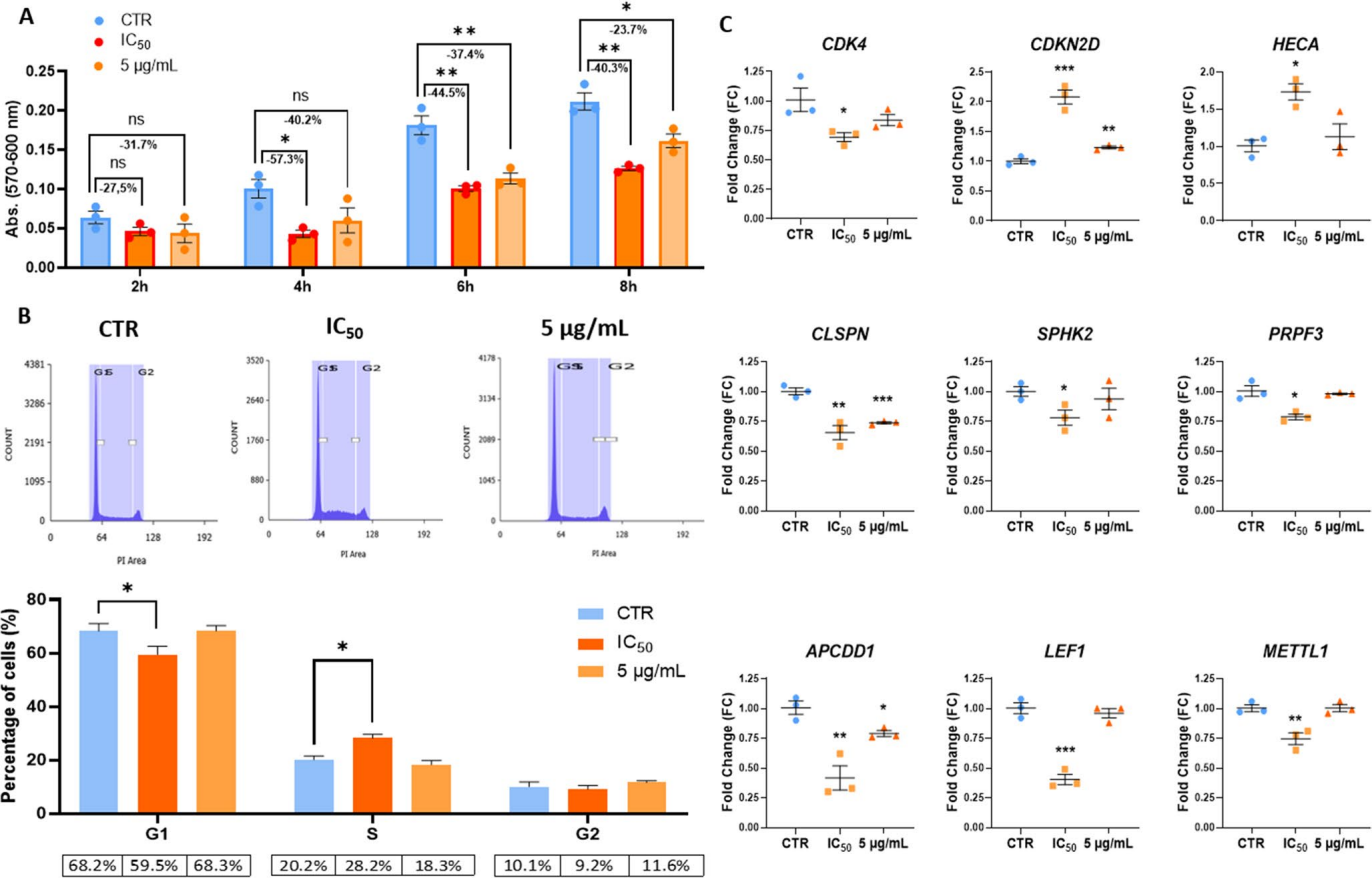
Proliferation-related effects of digested red grape anthocyanins in DLD1 cells. (**A**) AlamarBlue proliferation kinetics at IC₅₀ and 5 µg/mL; (**B**) Cell cycle phase distribution by flow cytometry; (**C**) RT-qPCR validation of selected proliferation-associated genes. *p < 0.05, **p < 0.01, ***p < 0.001 (t-test)

Since apoptosis alone did not fully account for the ~ 50% reduction in cell viability, antiproliferative effects were also investigated. AlamarBlue assays confirmed a significant decrease in cell number as early as 4 h post-treatment with the IC50 concentration, with a less pronounced effect at 5 µg/mL (Fig. 4A). Cell cycle analysis revealed a marked accumulation of cells in the S phase (Fig. 4B). RT-qPCR validation showed significant downregulation of key cell cycle promoters (CDK4, CLSPN, SPHK2, PRPF3, APCDD1, LEF1, METTL1) and upregulation of antiproliferative genes (CDKN2D, HECA), with dose-dependent modulation at 5 µg/mL (Fig. 4C).

Red grape polyphenolic extracts, including anthocyanins, have been shown to suppress CRC cell proliferation primarily through S and G2/M phase arrest and upregulation of cell cycle checkpoints [6, 22, 27]. These findings suggest that red grape polyphenols may disrupt DNA synthesis during the S phase, consistent with their ability to induce DNA fragmentation. Furthermore, red wine polyphenols have been shown to upregulate key regulators such as p53 and p21 (CDKN1A), supporting their role in enforcing cell cycle checkpoints in CRC [22]. Our results demonstrate for the first time that red grape anthocyanins retain these anti-proliferative properties following gastrointestinal digestion, maintaining the ability to arrest the cell cycle in the S phase. Treatment with the digested extract led to downregulation of CDK4, a cyclin-dependent kinase promoting the G1/S transition, and strong upregulation of p19/INK4D (CDKN2D), a tumor suppressor that inhibits this transition. Claspin (CLSPN), a facilitator of S-phase DNA replication, was inhibited, while HECA, a negative regulator of proliferation at the G2/M checkpoint, was upregulated. Several other positive regulators of proliferation in CRC, including SPHK2, PRPF3, APCDD1, LEF1, and METTL1, were also significantly downregulated.

### Digested Red Grape Anthocyanins Hamper Confined Cell Migration in DLD1 Cells

**Fig. 5:**
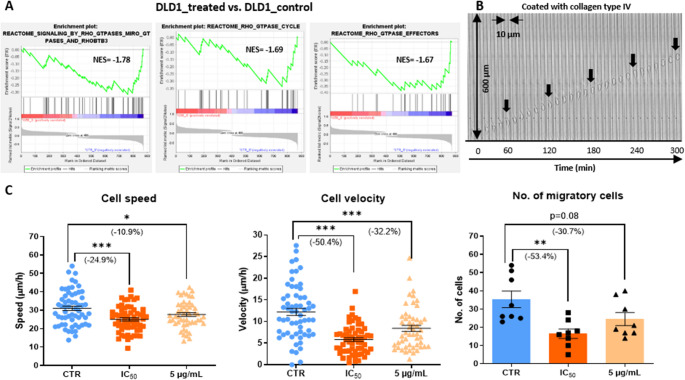
Cell motility-related effects of digested red grape anthocyanins in DLD1 cells. (**A**) Enriched motility-related Reactome gene sets (GSEA); (**B**) Spatial characteristics of the microfluidic device; (**C**) Confined 3D migration parameters (speed, velocity, migratory cell number) at IC₅₀ and 5 µg/mL. *p < 0.05, **p < 0.01, ***p < 0.001 (t-test)

GSEA analysis predicted that treatment with the digested red grape anthocyanin extract led to the inhibition of signaling pathways regulated by RHO GTPases, key molecular regulators of cytoskeletal dynamics and cell adhesion involved in cell migration (Fig. 5A). Additionally, the activity of MIRO proteins, critical for mitochondrial transport and intracellular distribution, was also predicted to be suppressed following treatment, suggesting potential disruption of cell polarity and impairment of directional migration (Fig. 5A).

To confirm that directional migration under 3D confinement is inhibited by treatment with the digested anthocyanins, migration assays were performed using microfluidic devices containing 10 μm wide channels (Fig. 5B). Treatment with the IC_50_ concentration of the digested anthocyanin extract significantly reduced the migration capacity of DLD1 cells, leading to a 24.9% decrease in migration speed and a 50.4% reduction in migration velocity, a parameter that reflects both speed and directionality over time (Fig. 5C). Additionally, the overall number of migratory cells was markedly decreased, with over 50% fewer cells entering the channels within the first 24 h of the assay after treatment (Fig. 5C). A significant reduction in migration was also observed at the lower concentration of 5 µg/mL based on all three parameters, although the effect was less pronounced (Fig. 5C).

Compared to their pro-apoptotic and anti-proliferative effects, the impact of anthocyanins on metastasis-related processes, such as epithelial-to-mesenchymal transition (EMT) and cancer cell migration, has been far less explored in CRC, with even fewer studies focusing specifically on red grape anthocyanins [6, 27]. Anthocyanins extracted from the grape family fruit *Vitis coignetiae* Pulliat have been shown to inhibit cell migration and invasion by downregulating claudins and matrix metalloproteinases (MMPs) [28] while a red wine polyphenolic extract reduced cell migration, EMT markers, and stemness-associated traits in CRC models [22]. On the other hand, no data are available regarding the effects of gastrointestinal digestion on the migrastatic activity of red grape anthocyanins or polyphenols in CRC. Although these findings provide important insights into the antimetastatic potential of grape-derived anthocyanins, the migration assays employed thus far do not accurately mimic the confined microenvironment and gradient cues present in vivo, lack single-cell resolution, and often rely on endpoint measurements, limiting their physiological relevance. Using 3D microfluidic systems, our results show that digested red grape anthocyanins markedly impair the confined migration capacity of CRC cells, with directionality, rather than speed, emerging as the most affected parameter. Transcriptomic profiling suggests that this migrastatic effect is primarily mediated through the suppression of signaling pathways regulated by RHO GTPases and MIRO proteins. RHO GTPases function as molecular switches, cycling between inactive GDP-bound and active GTP-bound states to orchestrate key aspects of cell motility, including actin polymerization, lamellipodia and filopodia formation, and cell contractility [29]. In parallel, MIRO proteins regulate mitochondrial transport and distribution by linking mitochondria to cytoskeletal motor proteins, thereby controlling their movement along microtubules, a critical process for maintaining cell polarity and directional migration [30].

From a translational perspective, although the IC₅₀ of 13.6 µg/mL observed for the digested extract cannot be directly extrapolated to dietary recommendations, the low systemic bioavailability of anthocyanins (< 1% of ingested dose) [8] does not preclude local anticancer effects within the gastrointestinal tract. Given that red grape cultivars typically contain 50–150 mg anthocyanins per 100 g fresh weight [31], luminal concentrations may reach micromolar levels through direct contact with colonic epithelial cells. Furthermore, colonic microbiota extensively catabolize residual anthocyanins into phenolic acids, including protocatechuic acid, which we identified at high concentrations post-digestion and which may significantly contribute to the observed bioactivity [32]. While our data support the biological plausibility of anthocyanin-mediated chemoprevention and suggest that digested forms may be more active than native compounds, validation in preclinical animal models and ultimately in randomized controlled trials is warranted before definitive dietary recommendations can be made.

## Conclusion

This study demonstrates that red grape anthocyanins can enhance their antitumor activity against colorectal cancer cells following simulated gastrointestinal digestion, as revealed by whole-transcriptome microarray profiling and validated by functional assays. The digested anthocyanins promoted apoptosis through both intrinsic and extrinsic pathways, suppressed cell proliferation via S-phase arrest and modulation of key cell cycle regulators, and impaired confined, directional migration by inhibiting RHO GTPase- and MIRO-mediated signaling. These multifaceted effects suggest the potential of digested anthocyanins as functional bioactives that merit further investigation for colorectal cancer management. By assessing post-digestion bioactivity, our findings provide a physiologically relevant framework for developing anthocyanin-based dietary strategies targeting colorectal cancer. However, the inherent limitations of in vitro models, including the lack of tumor–stroma interactions, immune surveillance, and the use of a single acute exposure, warrant validation in preclinical animal models and clinical trials before definitive dietary recommendations can be made.

## Supplementary Information

Below is the link to the electronic supplementary material.

Supplementary Material 1

## Data Availability

No datasets were generated or analysed during the current study.
