# Supplementary material for: Gastrointestinal Digestion Enhances the In vitro Antitumor Activity of Red Grape Anthocyanins Against Colorectal Cancer Cells
Source: Plant Foods Hum Nutr. 2026 Apr 14;81(2):46. doi: 10.1007/s11130-026-01498-w (PMC13079503; doi:10.1007/s11130-026-01498-w)
Supplement: Supplementary file 1 — Supplementary Material 1 [file 11130_2026_1498_MOESM1_ESM.docx]

**Gastrointestinal digestion enhances the in vitro antitumor activity of red grape anthocyanins against colorectal cancer cells**

**Daniel Cruceriu^1,2^, Denisa Dobos^1,2^, Stefan Miron^1,2^, Oana Baldasici^1^, Loredana Balacescu^1^, Ovidiu Balacescu^1^, Zorita Diaconeasa^3*^**

^1^ The Oncology Institute “Prof. Dr. Ion Chiricuta”, Department of Genetics, Genomics and Experimental Pathology, 34-36 Republicii Street, 400015 Cluj-Napoca, Romania

^2^ “Babes-Bolyai” University, Department of Molecular Biology and Biotechnology, 5-7 Clinicilor Street, 400006 Cluj-Napoca, Romania

^3^ University of Agricultural Science and Veterinary Medicine, Faculty of Food Science and Technology, 3-5 Calea Manastur Street, 400372 Cluj-Napoca, Romania

^*^Corresponding author. E-mail: [zorita.sconta@usamvcluj.ro](mailto:zorita.sconta@usamvcluj.ro%20) , Telephone: +40 751033871

**Supplementary data**

**Reagents** All chemicals were purchased from Sigma-Aldrich (Germany), unless otherwise specified.

**Anthocyanin extract preparation** Anthocyanins were isolated from red grape skins (Vitis vinifera). Polyphenols were extracted with acidified methanol (0.1% HCl) by sonication at 37°C for 5 min., followed by centrifugation at 5000 rpm for 5 min. and filtration using paper filters. The process was repeated until the plant material was fully depigmented. The pooled extracts were concentrated under reduced pressure at 41°C (Hei-VAP Expert, Heidolph, Germany), dissolved in acidified methanol (0.01% HCl), passed through 0.45 µm filters, and stored at -20°C.

Anthocyanin purification was performed by solid-phase extraction (SPE) on pre-activated Sep-Pak C18 cartridges. Sugars, acids, and water-soluble impurities were washed with acidified water (0.01% HCl), followed by removal of less polar polyphenols with ethyl acetate. Anthocyanins were then eluted with acidified methanol (0.01% HCl), concentrated, dissolved and filtered following the same procedures described for polyphenol extraction. The anthocyanin extract was stored at -20°C until use.

**Static *in vitro* digestion and purification of the anthocyanin extract**

Simulated gastrointestinal digestion was conducted using the INFOGEST 2.0 static protocol [9]. The extract was sequentially incubated in simulated saliva (SSF), gastric (SGF), and intestinal (SIF) fluids, with enzymes and cofactors added to each phase. The oral phase contained SSF with α-amylase (75 U/mL) and CaCl₂ (0.3 M). The gastric phase included SGF with pepsin (2000 U/mL), gastric lipase (60 U/mL), and CaCl₂, adjusted to pH 3. The intestinal phase contained SIF with pancreatin (equivalent to 100 U/mL trypsin), bile salts (10 mM), and CaCl₂, adjusted to pH 7. All incubations were performed at 37°C with agitation at 170 rpm.

Post-digestion, samples were centrifuged for 40 min. at 9000 rpm, the supernatant acidified with 0.5% formic acid, and centrifuged again for 10 min at 5000 rpm. Metabolites were purified by SPE (Sep-Pak C-18 cartridges). Salts were removed with acidified water (0.25% formic acid), and metabolites eluted with acetonitrile-formic acid (25%/0.25%). The eluates were concentrated at 41°C under reduced pressure, dissolved in acidified methanol (0.01% HCl), filtered, and stored at -20°C for analysis.

**Phytochemical characterization of anthocyanin extracts using HPLC-DAD/ESI+-MS**

Anthocyanins, phenolic acids, and flavonoids were analyzed using an Agilent HP-1200 HPLC system (Agilent Technologies, CA, USA) equipped with an autosampler, quaternary pump, diode array detector (DAD), and 6110 single-quadrupole MS detector. Separation was achieved on a Kinetex XB C18 column with dimensions of 4.6 × 150 mm and 5 μm particles (Phenomenex, USA) maintained at 25°C. The mobile phases consisted of water + 0.1% acetic acid (A) and acetonitrile + 0.1% acetic acid (B). The gradient elution program (0.5 mL/min, 30 min) was as follows: 0-2 min, 5% B; 2-18 min, 5-40% B; 18-20 min, 40-90% B; 20-24 min, 90% B; 24-25 min, 90-5% B; 25-30 min, 5% B. The injection volume was 10 μL. Mass spectrometric measurements were performed in positive ionization mode with an electrospray ion source voltage of 3000 V and a capillary temperature of 350°C. Data were collected in full scan mode within the m/z range of 280-1000. DAD spectra were recorded from 200-600 nm, with chromatograms monitored at 280 nm (phenolic acids), 360 nm (flavonoids), and 520 nm (anthocyanins). For quantification, calibration curves were constructed using commercial standards in the concentration range of 1-100 μg/mL. Anthocyanins were quantified as cyanidin equivalents (R² = 0.9951, LOD = 0.36 μg/mL, LOQ = 1.44 μg/mL). Phenolic acids were quantified as chlorogenic acid equivalents (R² = 0.9937, LOD = 0.41 μg/mL, LOQ = 1.64 μg/mL) and gallic acid equivalents (R² = 0.9978, LOD = 0.35 μg/mL, LOQ = 1.05 μg/mL). Data were processed using Agilent ChemStation software (v. B.02.01 SR2).

***Cell lines and culture conditions***

Two human colorectal carcinoma cell lines were used: Caco2 (ATCC, USA), cultured in MEM supplemented with 10% FBS, 1% penicillin-streptomycin, 1% L-glutamine, and 1% NEAA; and DLD1 (ECACC), maintained in RPMI-1640 with 10% FBS, 1% penicillin-streptomycin, and 1% L-glutamine. Cells were incubated at 37°C with 5% CO₂ and passaged at 80–90% confluence. All reagents were from Gibco, Thermo Fisher Scientific (USA).

***Evaluation of the in vitro antitumor activity by the MTT assay***

The in vitro antitumor activity of undigested and digested anthocyanin extracts from red grapes was evaluated using the MTT assay (#M6494, Invitrogen), following the manufacturer’s protocol. Briefly, Caco2 and DLD1 cells were seeded into 96-well plates at a density of 2 x 10⁴ cells/well. After a 24-hour incubation, the culture medium was removed and the cells were treated with serial dilutions of the extracts. The undigested extract was applied in eight concentrations ranging from 5 to 40 µg/mL, while the digested extract was tested in a narrower range of 1 to 25 µg/mL, based on preliminary experiments indicating increased antitumor activity of the digested form. Each concentration was tested in six technical replicates. Following a 24-hour treatment period, the supernatant was discarded, and 100 µL of MTT solution (1 mg/mL) was added to each well. After a 1-hour incubation, the MTT solution was removed, and 150 µL of DMSO was added to solubilize the formazan crystals. Absorbance was measured at 570 nm using a Synergy HTX microplate reader (BioTek Instruments, USA). All experiments were performed in biological triplicates.

Cell viability was expressed as the percentage of viable cells relative to untreated controls, based on absorbance values. Statistical significance of differences between treated and control groups was assessed using the t-test. The half-maximal inhibitory concentration (IC50) values for each extract and cell line were determined by nonlinear regression analysis (curve fitting) using GraphPad Prism Software Version 8 (GraphPad Software, USA). The relative potency of the digested extract compared to the undigested form was calculated as the ratio of their respective IC₅₀ values for each cell line. The Extra Sum-of-Squares F Test was used to compare dose-response curves and assess whether the IC₅₀ values of the digested and undigested extracts differed significantly.

***Validation of antitumor activity using the Trypan Blue exclusion assay***

The *in vitro* antitumor activity of the digested anthocyanin extract from red grapes on the DLD-1 cell line was validated by assessing relative cell viability using the Trypan Blue exclusion assay. Cells were seeded in 6-well plates at a density of 4 x 10^5^ cells/well in 2 mL of complete culture medium per well. After a 24-hour incubation, cells were treated with the digested extract at two concentrations: the IC_50_ concentration, determined previously by the MTT assay, and 5 µg/mL. The 5 µg/mL concentration was selected as it corresponded to approximately 75% cell viability compared to untreated controls, according to the MTT dose-response curve. After a 24-hour treatment, 10 µL of the cells suspensions from each condition were harvested and mixed in a 1:1 ratio with the Trypan Blue dye. The percentage of viable cells was determined using the EVE™ Automated Cell Counter (NanoEnTek Inc., Korea). All experiments were conducted in four independent biological replicates. Data were expressed as the relative viability of treated cells compared to the untreated control. Statistical analysis was performed using the t-test to compare treatment groups with the control.

***Evaluation of cell death by the Annexin V/PI assay***

The potential of the digested anthocyanin extract from red grapes to induce apoptosis or necrosis in DLD1 cells was evaluated using the FITC Annexin V/Dead Cell Apoptosis Kit (#V13242, Thermo Fisher Scientific), followed by flow cytometric analysis. In this assay, cells were treated with the extract at two concentrations: the previously determined IC_50_ and 5 µg/mL. Briefly, DLD1 cells were seeded in 6-well plates at a density of 4 x 10^5^ cells/well in 2 mL of medium and incubated for 24 hours to allow adherence. Cells were then treated with the extract or left untreated (control) for an additional 24 hours. After treatment, both adherent and floating cells were collected, washed with cold PBS, and resuspended in 1X Annexin Binding Buffer at a density of 1 x 10^6^ cells/mL. For staining, 100 µL of the cell suspension were incubated with 5 µL FITC-conjugated Annexin V and 1 µL propidium iodide (PI) for 15 minutes at room temperature, protected from light. After incubation, 400 µL of 1X Binding Buffer were added to each sample, and cells were immediately analyzed by flow cytometry using the S3e Cell Sorter (Bio-Rad, USA), equipped with 488 nm and 561 nm lasers. All experiments were performed in four independent biological replicates. Differences in the proportions of viable, early apoptotic, and late apoptotic cells between control and treatment groups were evaluated using t-tests.

***Evaluation of cell proliferation by the AlamarBlue assay***

The proliferation kinetics of DLD1 cells treated with the digested anthocyanin extract from red grapes was evaluated using the AlamarBlue assay (#DAL1025, Thermo Fisher Scientific), according to the manufacturer’s instructions. Briefly, cells were seeded in 96-well plates at a density of 1 x 10^4^ cells/well in 100 µL of culture medium, with five technical replicates per biological replicate. After a 24-hour incubation to allow cell adhesion, cells were treated with the extract at two concentrations: the IC_50_ and 5 µg/mL. Following treatment, 20 µL of AlamarBlue reagent were added to each well. Absorbance was measured at 570 nm and 600 nm at 2, 4, 6, and 8 hours post-treatment using a Synergy HTX microplate reader (BioTek Instruments, USA). Absorbance values at 600 nm from blank wells (containing only culture medium and extract) were used for background correction. All experiments were conducted in three independent biological replicates. Statistical significance of differences between control and treatment groups was assessed using the t-test.

***Evaluation of cell cycle progression by flow cytometry***

The effect of the digested anthocyanin extract from red grapes on cell cycle progression in DLD1 cells was evaluated by flow cytometric analysis of DNA content, following propidium iodide (PI) staining (#V13242B, Thermo Fisher Scientific). Cells were seeded in 6-well plates at a density of 4 x 10^5^ cells/well in 2 mL of culture medium and incubated for 24 hours to allow adherence. Subsequently, cells were treated with the extract at the IC_50_ concentration and 5 µg/mL, while untreated cells served as controls. After 24 hours of treatment, cells were harvested by trypsinization, washed with cold PBS, and fixed in 70% ethanol. Following fixation, cells were permeabilized with 0.1% Triton X-100 and stained with 50 µg/mL propidium iodide (PI) in a solution containing 50 µg/mL RNase A (#46-7604, Invitrogen). Samples were analyzed using the S3e Cell Sorter (Bio-Rad, USA), equipped with 488 nm and 561 nm lasers. Experiments were performed in three independent biological replicates. The distribution of cells across different cell cycle phases was compared between groups using paired t-tests to assess statistical significance.

***Evaluation of cell migration in 3D microfluidic devices***

The confined migration capacity of DLD1 cells treated with the digested anthocyanin extract from red grapes was assessed using 3D microfluidic devices fabricated as previously described [10]. The devices were produced by casting polydimethylsiloxane (PDMS; Dow Corning, Midland, MI) onto a microstructured mold created by standard photolithographic techniques. Each device consisted of a central well connected to the surrounding medium through two arrays of 60 parallel microchannels, each measuring 600 µm in length and 10 µm in width. Both the central well and the channels were coated with type IV collagen at a concentration of 20 µg/mL to promote cell adhesion and migration.

Following a 24-hour treatment with the digested anthocyanin extract at either the IC₅₀ concentration or 5 µg/mL, 4 x 10^4^ cells were seeded into the central well of the microfluidic device. The loaded devices were then placed on a BioStation IM-Q microscope (Nikon, Japan) equipped with an incubation chamber to maintain optimal culture conditions. Cell migration was monitored by time-lapse phase-contrast microscopy, with images acquired every 10 minutes over a 24-hour period using a 10X objective. All experiments were performed in four independent biological replicates.

Individual cell migration was analyzed using the Manual Tracking plugin in ImageJ software. For each biological replicate, the first 15 cells that entered the channels were tracked to determine their migration speed, defined as the average displacement per 10-minute interval, and their migration velocity, defined as the net displacement over the total distance traveled. The number of migratory cells was quantified as the total number of cells that entered an array of 30 microchannels during the 24-hour assay period. Differences in migration parameters between treated and untreated cells were assessed for statistical significance using the t-test.

***Transcriptome analysis by microarray expression profiling***

Whole transcriptome profiling was conducted using microarray technology on four biological replicates of DLD1 cells treated with the IC_50_ concentration of digested red grape anthocyanin extract, compared to untreated controls. Cy3-labeled cRNA probes were synthesized from 100 ng of total RNA using the Low Input Quick Amp Labeling Kit (#5190-2331, Agilent), following the manufacturer’s protocol. Probe quality and concentration were assessed using a NanoDrop ND-1000 spectrophotometer (Thermo Fisher Scientific) and the Bioanalyzer 2100 (Agilent). Hybridizations were performed for 17 hours at 65°C on Agilent Whole Human Genome Microarray 4x44k microarrays. Slides were scanned at 3 µm resolution using the Agilent G2505C Microarray Scanner, and raw data were extracted with Agilent Feature Extraction software (v11.5.1.1).

Data preprocessing and differential expression analysis were performed in R using the Bioconductor package *limma* [11]. Raw median signal intensities were quantile normalized across arrays, and summarized by computing the median of replicate probes. Control and flagged features were excluded. Differential expression was determined using linear modeling with empirical Bayes moderation. P-values were adjusted for multiple testing using the Benjamini-Hochberg method. Genes with an absolute fold regulation ≥ 1.5 and adjusted p-value < 0.05 were considered significantly differentially expressed. Microarray data are available in the NCBI Gene Expression Omnibus (GEO) under accession number GSE307005. To identify the main molecular mechanisms of action triggered by the digested anthocyanin extract from red grapes, Gene Set Enrichment Analysis (GSEA) [12] was performed using the Hallmark (h.all.v2024.1.Hs.symbols) and Reactome (c2.cp.reactome.v2024.1.Hs.symbols) gene set collections. Gene sets were considered significantly enriched at a false discovery rate (FDR) < 25% and nominal p-value < 0.05.

***Gene expression analysis by RT-qPCR***

To validate the microarray results, RT-qPCR was performed on randomly selected genes involved in apoptosis, cell proliferation and cell migration in DLD1 cells treated with the IC_50_ concentration of digested red grape anthocyanin extract. In addition, gene expression was evaluated in cells treated with 5 µg/mL of the digested extract to assess dose responsiveness. Following 24 hours of treatment, total RNA was extracted using the phenol-chloroform method after cell lysis with TriReagent (#AM9738, Invitrogen). RNA quality and concentration were assessed using a NanoDrop ND-1000 spectrophotometer (Thermo Fisher Scientific). For each sample, 500 ng of total RNA was reverse-transcribed into cDNA using the High Capacity cDNA Reverse Transcription Kit (#4368813, Applied Biosystems). Quantitative PCR was conducted using the LightCycler TaqMan Master Kit (#04735536001, Roche) on a LightCycler 480 instrument (Roche), following the manufacturer’s instructions. Primer and Universal Probe Library (UPL) probe sequences are provided in Supplementary Table 1. Relative gene expression levels were calculated using the ΔΔCt method [13], with RN18S1 used as the reference gene. All reactions were performed in three independent biological replicates. Statistical significance between treatment groups was determined using the t-test.

**Table 1. Identification and quantification of anthocyanins and their metabolites in undigested and digested extracts of red grapes**. Rt - retention time; [M+H]^+^ - molecular ion; UV λ_max_ - wavelengths of maximum absorption in the visible region.

| **Peak No.** | **Rt (min)** | | **UV λ_max_ (nm)** | **[M+H]^+^ (m/z)** | **Compound** | **Concentration (µg/ml)** | |
| --- | --- | --- | --- | --- | --- | --- | --- |
|  | **Before digestion** | **After digestion** |  |  |  | **Before digestion** | **After digestion** |
| 1 | 10.28 | 10.88 | 519, 281 | 465 | Delphinidin-3-O-glucoside | 232.521 | 0.000 |
| 2 | 11.15 | 11.15 | 520, 280 | 449 | Cyanidin-3-O-glucoside  Malvidin-3-O-caffeoyl-glucoside | 242.559 | 107.032 |
| 3 | 12.25 | 12.58 | 519, 279 | 655, 493, 463 | Malvidin-3-O-glucoside  Peonidin-3-O-glucoside | 254.210 | 128.954 |
| 4 | 15.62 | 15.92 | 519, 280 | 611 | Delphinidin-3-O-coumaroyl-glucoside | 165.482 | 40.478 |
| 5 | 16.40 | 16.67 | 518, 281 | 625 | Petunidin-3-O-coumaroyl-glucoside | 56.500 | 21.567 |
| 6 | 17.24 | 17.47 | 519, 279 | 639, 609 | Malvidin-3-O-coumaroyl-glucoside Peonidin-3-O-coumaroyl-glucoside | 65.104 | 24.758 |
| **Total anthocyanins** | | | | | | **1016.376** | **322.789** |
| M1 |  | 3.71 | 270 | 301 | p-Hydroxybenzoil-glucose | 0.000 | 71.520 |
| M2 |  | 4.64 | 270 | 171 | Gallic acid | 0.000 | 92.120 |
| M3 |  | 6.65 | 270 | 155 | Gentisic acid | 0.000 | 101.430 |
| M4 |  | 9.55 | 280 | 317 | Protocatechuic acid-glucoside | 0.000 | 25.720 |
| M5 |  | 9.72 | 290 | 155 | Protocatechuic acid | 0.000 | 110.364 |
| M6 |  | 13.61 | 280 | 169 | Vanillic acid | 0.000 | 60.370 |
| M7 |  | 13.99 | 310 | 165 | *p*-Coumaric acid | 0.000 | 74.880 |
| M8 |  | 16.06 | 326 | 195 | Ferulic acid | 0.000 | 71.200 |
| **Total metabolites** | | | | | | **0.000** | **607.604** |
